# Co-Creation Technology Development of a Speech Therapy Platform in Germany: Results from an Exploratory Usability Workshop

**DOI:** 10.63144/ijt.2026.6764

**Published:** 2026-06-01

**Authors:** Katharina Giordano, Lea Haas, Jasmin D M Pfannes, Julia Hagenow, Juliane Leinweber

**Affiliations:** HAWK University of Applied Sciences and Arts, Hildesheim/Holzminden/Göttingen, Göttingen, Germany

**Keywords:** Co-creative methods, Participatory-design, People with speech and/or language impairment, Technology development process, Telepractice, Therapy platform

## Abstract

Telepractice as a service model in speech and language therapy has significantly grown in Germany since the pandemic. However, the implementation is not yet satisfactory. The Hybrid and Interactive Speech and Language Therapy after Stroke project (HiSSS) aimed to provide a complex therapy platform that would integrate both synchronous and asynchronous therapeutic elements for the first time in Germany. This study aimed to evaluate the usability of an early-stage platform by involving people with speech and/or language impairment and speech and language therapists in the development process to ensure that the system was tailored to their needs. Structured observation and a focus group were used in an exploratory usability workshop. Participants provided feedback on facilitators and barriers, suggestions for improvement, training needs, and support while using the platform. The results of the exploratory usability workshop showed that both end user groups would be interested in a future use of the HiSSS platform.

The lifetime prevalence of stroke among adults in Germany was approximately 3% between 2017 and 2022. Prevalence increased significantly with age, reaching 7.5% among those aged 65–79 (Robert Koch-Institut, 2024). These figures are consistent with comparable international statistics (Rajati et al., 2023; Tsao et al., 2023). Due to the wide range of consequences associated with stroke, it is one of the leading causes of adult disability (Lozano et al., 2012). Common consequences include speech impairments such as dysarthria and apraxia of speech, and language impairments such as aphasia, which can occur individually or in combination (Mitchell et al., 2021; Schindel et al., 2022). These impairments affect communicative interaction and thus have a direct impact on the everyday lives of those affected. This often leads to social isolation (Dickson et al., 2008; Nätterlund et al., 2010), impairs psychological wellbeing, and decreases quality of life (Lam & Wodchis, 2010).

Speech and language therapy aims to counteract the negative consequences of stroke by alleviating symptoms of speech and language disorders and their associated psychosocial effects. Research confirms the effectiveness of interventions that improve functional outcomes such as reading, writing and expressive language (Brady et al., 2016). Individuals appear to benefit particularly from high intensity, high dose treatment (Breitenstein et al., 2017). However, high-frequency therapy often cannot be implemented due to health policy limitations, a shortage of specialists, and limited patient resources (Aston et al., 2025; Brady et al., 2016). In recent years, telepractice has gained popularity as a means of improving access to speech and language therapy. Telepractice uses information and communication technology to provide rehabilitation services remotely. The technology connects healthcare providers with beneficiaries to perform screening, assessment, intervention, counselling, and/or monitoring (Hill et al., 2025).

Telepractice can be conducted synchronously or asynchronously. Synchronous telepractice usually involves an exchange via video conference with a therapist, either individually or in a group setting. In contrast, synchronous telepractice involves providing information or material that enables patients to perform exercises independently, without the therapist’s simultaneous support. Different forms of telepractice can be combined with each other, as well as with in-person services. Service delivered via telepractice is primarily intended to complement, rather than replace, standard in-person care. Studies conducted internationally confirm that telepractice is a feasible and effective treatment for speech and language impairments (Cetinkaya et al., 2023; Cordella et al., 2022; Øra et al., 2020; Teti et al., 2022). Though telepractice had already been established in speech and language therapy internationally for some years (e.g., Teti et al., 2023; Weidner & Lowman, 2020), it has significantly grown worldwide (Hilari et al., 2024). Telepractice received regulatory status in the health service in Germany for the first time during the COVID-19 pandemic (Wittmar et al., 2023). However, there is currently no technology in Germany that allows for the flexible combination of in-person service, asynchronous telepractice, and synchronous telepractice.

To address this need, Hybrid and Interactive Speech and Language Therapy (HiSSS)^1^ is envisioned as via complex therapy platform that can integrate both existing and innovative synchronous and asynchronous therapeutic elements. These innovative elements can include automatic speech recognition (ASR) and automatic face recognition (AFR) via the user’s device sensors, as well as the analysis results in therapy. All elements can be parts of speech and language therapy interactions and could be used during in-person therapy, video therapy sessions, and asynchronous, self-administered exercises.

The platform for HiSSS was developed through a co-created technology development process, engaging people with speech and/or language impairment (PWSLI) and speech and language therapists (SLT) (Giordano et al., 2025). Participatory technology development aims to create technologies that meet users’ needs by involving them in the development process and drawing on their knowledge of practices, context, and cultures, as well as health and illness. The goal is to develop user-centered solutions that are practical, sustainable, and tailored to the specific needs of the target groups (e.g., Spelter et al., 2022).

The development process was based on a co-creation approach (Vargas et al., 2022) and resulted in a first version of the HiSSS platform that was tested by users in an exploratory usability workshop within a co-creation setting. Because technology co-creation is a complex, usually agile process, it involves various disciplines (Tessarolo et al., 2019). Therefore, a focus group was additionally conducted to facilitate the dialogue between platform users and platform developers. Understanding the challenges faced by therapists and patients when using the HiSSS platform in an early stage would provide early insights into usability barriers and training needs.

This co-creation usability study focused on an early-stage platform evaluation with PWSLI and SLT to obtain preliminary usability impressions. Specifically, we explored potential barriers such as interface clarity, support needs of users, and speech-recognition problems.

## Methods

The study design, an exploratory usability workshop in a co-creation setting, included two parts: (1) a structured observation while testing the platform and (2) a focus group. The workshops were coordinated by the Speech and Language Therapy Lab at the HAWK in Göttingen. Ethical approval was received from the ethics committee of the HAWK University of Applied Sciences and Arts Hildesheim/Holzminden/Göttingen (01062022).

### Procedure

Two workshops were conducted back-to-back: the first for PWSLI, the second for SLT. Platform developers (engineers, designers and SLT) were guests to observe HiSSS and participated in the focus group.

The time schedule was identical for both workshops, with variations solely in the content. Adjustments were made to meet the needs of each participating group. For example, the presentation used to introduce the HiSSS-project to the group of PWSLI used simple language and additional illustrations. Each workshop had a planned duration of 2.5 hours. A detailed timeline can be found in Table 1. The workshop for PWSLI took place in the early afternoon and the workshop for SLT in the early evening.

The workshop took place in the Speech and Language Therapy Lab at the HAWK in Göttingen. Four different rooms were set up for the structured observation, and a main room was used for the welcoming session and introductions, as well as the focus group and a final closing session.

**Figure 1:**
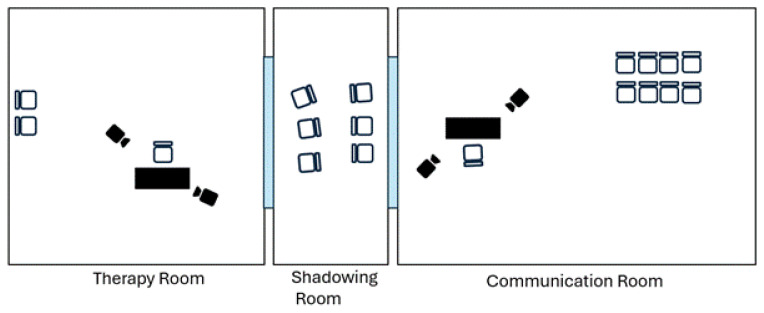
Setup for the Structured Observation in the Therapy and Communication Rooms

Observation sessions took place in a one-on-one setting, one session per room. The goal was to reduce distraction throughout the observation and create an ideal environment for video and audio recordings during the sessions. The setup in the rooms is illustrated in Figure 1 (Therapy Room, Shadowing Room and Communication Room). Cameras were positioned in each room to completely capture the participant as well as the tablet screen and the participant’s use of the tablet. Participants were accompanied by a previously trained researcher throughout the observation. A space was set up outside of the recorded area for the accompanying employee, who also had access to a list outlining the tasks to be completed during the observation. The participants were directly observed from a separate, shadowing room by platform developers or relatives of the participants (Shadowing Room; see Figure 1*)*. The participants were informed about the observing parties.

After completing the previously set tasks the participants were able to take time to explore the HiSSS platform independently. The researcher accompanying the observation remained available for questions during this explorative phase. The phase was also recorded on video.

After completion of the structured observations all workshop participants met for a break. The workshop break was intended for participants to get to know each other while also providing the opportunity to exchange ideas with the platform developers.

The focus group was conducted with platform users and platform developers. For the focus group, nine chairs were set up in a circle, four for the users and four for the developers and one for the session moderator. Materials such as flip chart papers, pens and moderation cards were made available to participants when necessary. The focus group was recorded with the cameras integrated in the room. The remaining platform developers and relatives of PWSLI were able to observe the focus group from the shadowing room.

After completion of the focus group the workshop was formally concluded.

### Participants

Four participants were recruited for each workshop: One group was consisted of four SLT, the other of four PWSLI. Participants for the PSWLI group were included if they were at least 18 years old, had experienced a stroke at least six months post-onset and were living with chronic speech and language impairment. Participants for the SLT group were included if they were speech and language therapists and were experienced in the treatment of patients with speech and language impairment. Participants in both groups were required to have experience with telepractice. Participants were recruited from an existing network of prior workshops and research projects and speech and language therapy practices between February and May 2024. In the case of no responses during the first weeks of the recruitment, additional participants were recruited by broadening the criteria to ensure the sample size and dataset for analysis. The medical diagnosis of stroke was not relevant for the early-stage platform evaluation. Therefore, the criteria for medical diagnosis inclusion were expanded to neurological diseases such as Parkinson Disease, brain injuries and haemorrhages.

Following the independent signing of consent forms, all participants completed a demographic questionnaire. A questionnaire about their technical affinities (Karrer et al., 2009) and use of technology in their everyday life (Mollenkopf et al., 2000) was used to ensure diverse experiences with technology among the groups. The PSWLI with language impairment were screened using BIAS-R (Richter & Hielscher-Fastabend, 2018). The PSWLI with speech impairment were screened with BODYS (Ziegler et al., 2018). This allowed for a detailed description of the individual impairments and signalled any needs for language adjustments during the workshops, to ensure comprehension.

Tables 3 and 4 provide the collected demographic data (age and gender) for each of the four participants in each workshop. A technological affinity score was calculated according to Karrer et al. (2009). Additionally, Table 2 lists the relevant speech and language impairment diagnoses for this group, diagnosed using the aforementioned screening tools. Table 3 shows the number of years of relevant working experience for each therapist at the time of the workshop. All recruited participants were involved in both parts of their respective workshops (the observation and the focus group).

### Data Collection

The aim of the workshop was to evaluate the preliminary usability of the early-stage HiSSS platform by involving end users in the development process and ensuring that the system was tailored to their needs. The evaluation focused on the usability impressions with the platform within a co-creation setting and combined structured observation (Boman et al., 2007) with a focus group discussion (Flick, 2012).

#### Structured Observation

Structured observations employed predefined evaluative checklists. Observation is an established and well-known method in speech-language pathology research (Cordes, 1994) and has previously been applied in usability research (Boman et al., 2007). Each participant individually tested the platform in a technology testing room, accompanied by a speech and language therapist as an observing researcher. Participants used either a tablet or laptop running the HiSSS platform and were assigned seven tasks that reflected typical use and covered the system’s major functionalities. This task-based usability assessment was predetermined through consensus by the platform developers and researchers. All possible solution paths were identified in advance, enabling both real-time support and retrospective analysis of user strategies. Differences in the solution paths between the application on the tablet and the web interface on the laptop were identified and considered (see Table 4). If participants had questions or were unable to complete a task, the observing researcher assisted. Sessions were video recorded.

Each reaction to a task was rated by the observing researchers on a prepared checklist with a four-point scale, ranging from “feasible without issues” to “not feasible (assistance in every step)” (see Table 5). Additional observations were documented in a comment section by the observing researcher. The checklist enabled analysis of effectiveness (rate of successfully completed tasks) and efficiency (level of user autonomy in task completion) of the HiSSS platform use.

#### Focus Group

Focus groups are moderated group discussions on a specific topic and have been used successfully in the evaluation of the usability of e-Health applications (Maramba et al., 2019). Despite communication challenges, focus groups are feasible for PWSLI (Azevedo et al., 2023). In-person formats enable the integration of nonverbal communication, supporting interaction and generating new perspectives (Azevedo et al., 2023). For individuals with aphasia, several factors have been identified to positively influence group situations and to be considered in the study design (Lanyon et al., 2018): balanced and equitable group interaction; open, non-hierarchical environment; communication awareness and mutual respect; meaningful goals and activities; structured process; appropriate group size and composition; and, professional facilitation and support.

Each participant in both groups (i.e., SLT and PWSLI) joined a focus group with the perspective of platform users. Additionally, platform developers joined the focus group with each platform user group. Bringing together users and developers (stakeholders) is meant to ensure the successful integration of technology in the future (Sanders & Stappers, 2008). A researcher moderated the sessions, which centered on user satisfaction with the HiSSS platform. They facilitated discussion and exchange among group members concerning feedback, questions, and answers. Guiding questions, derived from validated questionnaires (System Usability Scale (SUS), Brooke, 1996; Questionnaire for User Interface Satisfaction (QUIS), Chin et al., 1988; Usefulness, Satisfaction, and ease of use (USE), Lund, 2001; Telehealth Usability Questionnaire (TUQ), Parmanto et al., 2016), addressed topics such as: personal perceptions of using the HiSSS platform; willingness to use it in the future; aspects liked/disliked; and user emotions and attitudes during system use. The moderator ensured equitable participation and encouraged interaction among group members. The session was video recorded and lasted one hour.

### Data Analysis

#### Structured Observation

Observation checklists for each session were independently completed and analysed by two raters. The raters did not participate in the initial task observation and used the video recordings to evaluate the participants’ task performance using the four-point-scale of the checklist. If participants solved the task without assistance, their performance was rated as 1 or 2, depending on whether they solved the task immediately and confidently (rating of 1), or attempted alternative solution paths before succeeding (rating of 2). If the participants required assistance, their performance was rated as 3 or 4. A rating of 3 was assigned if participants were able to continue independently after receiving minor help, while a rating of 4 was assigned if they needed assistance at every step of the solution process. Additionally, the comment section was used throughout the evaluation to provide a detailed account of task completion or other unexpected events and comments made by the participants. After both raters had completed the checklists independently, their assessments were compared and any discrepancies were resolved through discussion and reviewing the video recording of the session to reach consensus.

The ordinal task ratings across the eight heterogeneous tasks were determined using the median for each group. Additional comments from the observation checklist were analysed inductively using qualitative content analysis (Elo & Kyngäs, 2008) to identify underlying causes when participants were unable to complete tasks independently or required substantial effort.

#### Focus Group

Central sections of the focus groups were orthographically transcribed and analysed using inductive content analysis (Elo & Kyngäs, 2008), guided by the research question of how potential users evaluated the HiSSS platform in terms of usability. The inductive process included identifying relevant text segments, grouping them into categories, defining categories and summarizing them into main categories. The content analysis was conducted by the first author. To ensure the quality of the data analysis, precautions based on the quality criteria of Lincoln and Guba (1985) were taken, including regular debriefing sessions and review of coding and category development between the first and last author, to strengthen the credibility and plausibility of findings. The data were managed using MAXQDA 2022 for Windows. All quotations cited here were translated from German into English; pauses, duplications and mispronunciations were corrected.

## Results

### Structured Observation

Observations were conducted to document the success of participants in solving researcher-assigned tasks in the HiSSS platform. The task-based usability assessment was conducted with PWSLI (P, n = 4) and SLT (T, n = 4). Eight tasks were evaluated by two raters: *send message*, *start training*, *open statistics*, *start video-call*, *write on whiteboard*, *end video-call*, *change settings* and *operator handover*. Each group was to perform seven tasks depending on their user perspective (PWSLI or SLT). Feasibility was rated on a 4-point scale (1 = feasible without issues, 2 = feasible after some attempts/after short time, 3 = feasible with assistance/after long time, 4 = not feasible/assistance in every step, X = not executed). Key patterns observed by the two raters are presented in Table 6. Among raters, there were discrepancies on only one task each for P1 and T4. Rater 1 scored in the task “write on whiteboard” for P1 a 2, while rater 2 scored a 3. Consensus was a rating score of 3. In task “start video-call” rater 1 scored a 2 and rater 2 scored a 1. In this case, consensus was a rating score of 2. Missing entries (X) were imputed by calculated median.

Across the tasks, the averaged Median of feasibility was lower for SLT (Median_alltasks_ = 1.64) than for PWSLI (Median_alltasks_ = 2.4). For PWSLI, the most feasible tasks were *open statistics* (P_Median_ = 1.5) and *start video-call* (P_Median_ = 2.0); *start training* (P_Median_ = 2.0) and *change settings* (P_Median_ = 2.0) were moderately feasible. *Send messages* (P_Median_ = 3.0) and *write on whiteboard* (P_Median_ = 3.5) was feasible with assistance. For participant P1 and P2 the tasks *start video call, end video call* and *change settings* were easily feasible rated. While for participant P3 and P4 these tasks were scarcely feasible rated (P3 = 4, P4 = 3–4). The task *operator handover* was only assessed for SLT and showed moderate feasibility (T_Median_ = 2.0); the task *start training* was only assessed for PWSLI and was moderately feasible (T_Median_ = 2.0). Overall, the SLT required less assistance to complete the tasks and were therefore able to complete the tasks independently, but two tasks resulted in missing values by half of the participants. Simultaneously there was a range of outcomes within the groups, emphasizing the individuality of the results.

In the next step of the analysis, underlying factors for the lack of independent task completion could be identified based on the video recordings. Three recurring themes emerged from the data including effectiveness, efficiency and setting. The first major theme identified was *effectiveness*. Participants described via think-aloud the malfunctioning of technology (e.g., speech analysis) as well as a lack of core functions/fundamental functioning (e.g., screen-sharing unrecognized) as reasons for unsuccessful completion of tasks. Another recurring theme was *efficiency*. It was characterized by named problems with the operation (e.g., buttons not visible) and design (e.g., suboptimal choice of colour) of the system, as well as with the conceptualization of the situation (e.g., unclear instructional design of tasks) that led to failed attempts to solve the tasks. The third theme identified was *setting*. Participants named the user device itself (e.g., typing was considered difficult) and the internet connection as factors for being unable to solve the tasks autonomously.

### Focus Group

The participants reported their individual perception and satisfaction with the HiSSS platform as well as suggestions for improvement. The researchers identified six main categories (Table 7).

#### Facilitators

This category refers to elements that stood out positively for the participants. The results show that participants were impressed by the technology, especially by the educational, motivational and exciting character of the system. Participants also named the variety of exercises and the combination of the video-call and the exercises: *“I thought it was cool it was so diverse [...], that it had so many possibilities”* (T3). In this category SLT described the customizability of the system: *“I was surprised how much I could adjust. [...] that was very good”* (T2), while PWSLI highlighted that the system was perceived as support to a person’s autonomy: *“I can pull myself out of the mud and for me that is [...] exciting and I am happy afterwards”* (P3).

#### Barriers

Participants identified barriers such as the inability to find certain functions and items being too small, as shown in the following comment: *“on this side the gear, the settings, I did not recognize it. Too small”* (P3). Additionally, certain features of the system were negatively perceived, such as time lags for the speech assessment tool and overlapping windows: *“I had to move it [the window] around constantly and sometimes it did not work with the swipe”* (T4). Furthermore, several in the SLT group described that they missed settings for the individual training-sets.

#### Suggestions for Improvement

Participants wished for additional support within the system, as examples: a description for the exercises, and further support options while completing the exercises. A separation between the video-call-window and the exercise window was preferred: *“I would like to have that [the windows] clearer”* (T4); *“displayed with two large tiles”* (T1). Many participants also wished for clearer instructions for the exercises.

#### Future (Non-)Use

SLT considered the system a useful supplement for independent training and both speech therapy and language therapy, and considered possibly using the system in the future: *“I think the idea is good especially considering the shortage of therapists”* (T3)*; “it is good to move forward [in the way therapy is provided] and to develop further”* (T2). Furthermore, the possible advantages for relatives of PWSLI and the integration in video-therapy were named. Some SLT were dismissive when considering using the system in a group setting: *“For now I could imagine it better in a 1-to-1 setting, because if you have many patients all trying to say something at the same time [...] I think you do not have control to lead it”* (T2).

#### Training and Support

For this category participants gave feedback on possibilities for support in the system. They especially described familiarization and good instructions as an important factor in the use of the system. The majority of the participants supported the idea of a guide or tutorial, guiding users through the system (“*something step by step at the start”* (T1)*; “what I would consider helpful is a guide at the start [...] a tutorial”* (T4)*; “a guide would be helpful to know where to find what”* (T1). There were various opinions on what type of guide would be useful. While some participants supported the idea of a user manual, video tutorial or digital assistant, other participants dismissed the same type of supporting materials: *“I would not use a manual”* (T2), *“intuitively I would not click on a video”* (T4).

#### Participation

While the participants consisted of both PWSLI and SLT, only PWSLI described an additional positive feeling towards the development of the system in terms of participation: *“I am hopeful that it picks up speed, that is a positive development”* (P2). PWSLI expressed their satisfaction regarding the prospect of making a contribution: “*I am very happy that we are here”* (P3). They also expressed trust in the project team, to further improve the system: *“for me that is a thing that the specialists can solve”* (P2). Participants explicitly wished for a recurring exchange in the developing process: *“When the group that is now working here with the laypersons. When they receive the information once it is ready, such an exchange might also be useful”* (P2) and offered to act as a multiplier for the system within their community: *“I am in a self-help group, and I could imagine acting as a multiplier”* (P2).

## Discussion

This preliminary study aimed to evaluate the usability of the early-stage HiSSS platform to integrate both existing and innovative synchronous and asynchronous therapeutic elements, for the first time in Germany. It also aimed to positively influence acceptance of the platform and, consequently, its long-term use as future platform. A combined approach of structured observation and a focus group was used to collect data on preliminary usability impressions of the platform’s effectiveness, efficiency, and satisfaction as well as facilitators and barriers to its use from the perspectives of PWSLI and SLT.

The results showed that SLT could generally complete the assigned tasks independently, they were unable to execute some tasks. However, there were more individual differences among PWSLI, with two out of four participants unable to complete the tasks independently and without assistance.

In terms of efficiency regarding the level of user autonomy in task completion, it was found that participants in both user groups encountered problems that prevented them from completing the tasks quickly and efficiently. These included buttons not being recognised as such, an unfavourable choice of colours within the system which made it difficult to use, as well as ambiguous or overly abstract labels. The subsequent focus group confirmed these findings regarding effectiveness and efficiency, and provided suggestions for improvement (e.g., standardised terminology and increased support).

As per the results of the focus group with respect to satisfaction of the HiSSS platform, all participants of both user groups expressed an interest in using the platform in the future. They considered the system as offering added value, particularly in supporting self-training and better integrating video therapy into standard health care service. However, the SLT were divided on its possible applications in group therapy, which were not explicitly evaluated in this early-stage platform evaluation. Platform users expressed that suitable instructional material in the form of an instructional video, frequently asked questions (FAQs) or telephone support would increase satisfaction.

A final category of results from the focus group, derived exclusively from the perspective of PWSLI, focused on participation. This category revealed the participants’ positive perception of co-creative methods of participatory design: they were involved in the technology development process as active and equal partners (Rießenberger & Fischer, 2023).

These preliminary efficiency results seem to be consistent with other literature and the previous requirements analysis for the HiSSS platform (Giordano et al., 2025). Large buttons, a high-contrast design, and technical support are frequently cited as requirements for intuitive technological systems (Hill et al., 2009; Øra et al., 2018; Weidner & Lowman, 2020). However, these are very general terms for which there are various implementation options. Similar issues have already arisen in other technology development projects, for example, discussions around what constitutes a ‘large font size’ (Spelter et al., 2022). The results of this preliminary co-creation usability study suggest the importance of regularly involving users iteratively, in order to evaluate an early-stage platform of initially identified requirements from the user’s perspective. The long-term goal of this preliminary usability evaluation was to investigate the general acceptance of the HiSSS platform to strengthen the probability of its sustainable use in a final development process.

**Figure 2:**
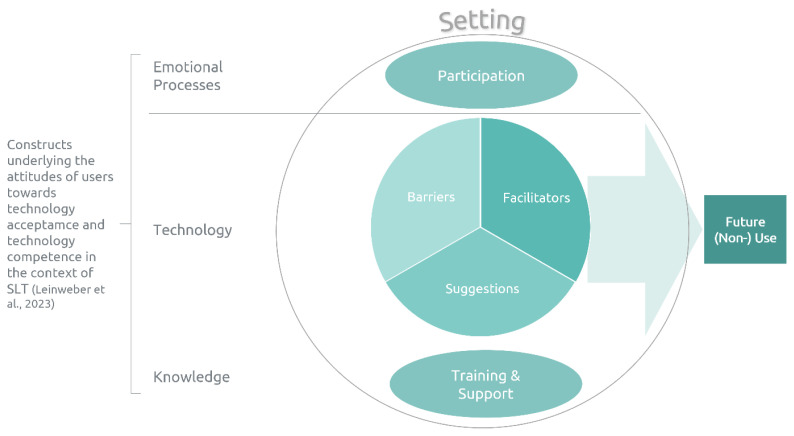
Mapping of the Usability Evaluation Results to the Factors Identified by Leinweber et al. (2023)

Technology acceptance is a complex construct resulting from a multi-stage decision-making process, which may lead to discontinuation of use at any stage (Hastall et al., 2017). In their analysis of the readiness to use video therapy in Germany, Leinweber et al. (2023) identified four influencing factors: (1) technology; (2) emotional processes; (3) knowledge; and (4) environment. The results of the current exploratory usability workshop could be assigned to the aforementioned factors (see Figure 2). The advantages and disadvantages mentioned by the participants, as well as the suggestions for additions to the HiSSS platform, could be attributed to *technological factors*. *Emotional processes* seemed to play a role in the ‘participation’ category, where participants expressed positive views about having the opportunity to actively contribute to the development process. By their suggesting that they may act as multipliers, the participants seemed to demonstrate a sense of ownership over the HiSSS platform and its development process. The *knowledge factor* could be reflected in the preliminary usability impressions when participants asked for customised, specific support and instructional material to enable them to use the HiSSS platform independently. *Environment-related* results might be indicated by the structured observations, when operating problems with the device or an unstable internet connection made using the early-stage platform difficult. All reported results provided preliminary usability impressions about participants’ assessment of future use or non-use. The reported results of the exploratory usability workshop suggest an acceptance of the HiSSS platform in future development. At the same time, it is clear that the ongoing process will also require the regular, iterative involvement of user groups, in order to evaluate adjustments to the platform from a user perspective. This approach supports further development of co-creation practices.

In contrast, questionnaires are the most frequently used approach and provide an overall measure of usability (Maramba et al, 2019). But they often fail to capture specific barriers and usability challenges faced by users. Consequently, mixed methods approaches in usability evaluation have become more prevalent and were adopted for the usability evaluation of the HiSSS platform in this exploratory usability workshop study. In particular, the usability evaluation of the HiSSS platform combined structured observation (Boman et al., 2007) with a focus group discussion (Flick, 2012). Similar methodological combinations have already been used for the usability assessment of technologies for individuals with speech impairments (DeCock et al., 2021; Frieg et al., 2017; Spelter et al., 2022). Observation is particularly suitable for evaluating effectiveness and efficiency while user satisfaction is generally assessed through qualitative methods such as interviews or focus groups.

## Limitations

### Recruitment Barriers

The project included a risk management plan to ensure that the project objectives were achieved as effectively as possible. An identified key area of risks was participant recruitment. A management plan was developed to define mitigation strategies and courses of action for future implementation if necessary. Since the recruitment did not go as well as expected, an expansion of recruitment efforts was planned in order to reach the target sample size. This involved broadening the inclusion criteria “medical diagnosis” from only stroke to neurological diseases.

### Small-n workshop study

Given the small sample size with n=8 due to the recruitment challenges discussed above, our findings must be interpreted with caution. Faulkner (2003) showed that the greater the number of participants, the greater the probability of identifying all relevant errors and problems in a usability evaluation. In this study, this workshop was just one part in an iterative design process and focussed on qualitative insights as to why users struggle with the platform. From an ethical perspective, resources should be used sparingly when working with a vulnerable group.

### Missing task observations

The structured observation seemed to be a useful method for an initial exploration of the systems usability. However, due to technical problems not all planned tasks could be carried out with all participants (see “X” in Table 6). For that reason, different numbers of participants were able to be included in evaluation of the tasks.

### Social desirability bias

Platform developers and a researcher were part of the focus group. Both are members of the project team and could provoke possible bias of social desirability. This means that when conducting the focus group platform, users might give socially desirable responses instead of responses that are reflective of their true feelings. In addition, when the researchers of the project team evaluate the focus group, socially desirable interpretation might happen. This might result in a more positive interpretation for the presented platform and hinder the further development process. To reduce the potential of social desirability, researchers attempted to create a climate of trust and used combined methods (group and individual interaction). The moderator framed non-judgmental questions (Grimm, 2010).

### Participant profile

The SLT group was homogeneous in age and working experience. One reason for this might be that the willingness to participate in the workshop correlated with the therapists’ background regarding their experience with video therapy (technical equipment, prior use, technology acceptance). This in turn could have led to a potential self-selection bias and an inaccurate self-assessment of their contribution to the workshop, due to telepractice being a new health-service since the pandemic (Wittmar et al., 2023). The PWSLI group were all male. This might be a problem, as the development of the HiSSS platform can run the risk of being gender-specific and more directed towards men (Rießenberger & Fischer, 2023).

## Conclusions

The results provide early insights into usability barriers such as missing training sets, overlapped windows, as well as the need for training and support while using a telepractice platform currently under development. In the next development process the HiSSS platform will be subject to co-creative methods in order to address the needs of the target group. There are diverse challenges when putting these methods into practice. In a long-term view the platform must be tested in end-user groups outside of the co-created technology development process. Specifically, its feasibility and acceptability as a potential teletherapy platform in the German health care service must be evaluated.

## Figures and Tables

**Table 1:** Detailed Timeline of the Usability Workshops

Agenda Item	Duration	Execution
Welcoming session	5 minutes	Researcher welcomed participants, relatives and platform developers (engineers, designers and SLTs).
Introduction of the HiSSS-project	15 minutes	Participants are introduced to the researcher team, the project goals and the goals of the technological developments.
Structured observation	30 minutes	Participants tested the HiSSS platform in a one-on-one setting by completing a previously developed list of tasks and using the think-aloud method to comment on their progress and convey first impressions.
Individual exploration of the platform and break	30 minutes	Participants individually explored HiSSS. Snacks and drinks are provided during the subsequent break. An exchange of ideas with platform developers and among the participants is encouraged.
Focus Group	60 minutes	PWSLI, SLT and platform developers take part in a focus group. A researcher moderates the focus group.

Closing session	10 minutes	The research team, platform developers, and participants gather to close the workshop. All attendees are thanked for their participation and contributions.

**Table 2:** Demographic Data for Participants with Speech and/or Language Impairment (P)

	P1	P2	P3	P4
**Age (years)**	54	75	72	64
**Gender**	male	male	male	male
**Diagnoses**	Aphasia (severe)	Dysarthria (mild)	Aphasia (mild)	Aphasia (moderate)
**Technological affinity** *	75	47	64	77

*Note*.

* Measured with TA-EG (Karrer et al., 2009): score ranges from 19 (low affinity for technology) to 95

**Table 3:** Demographic Data for Therapist (T)

	T1	T2	T3	T4
**Age (years)**	26	25	29	26
**Gender**	female	female	female	female
**Years of experience**	2	2	7	0.5
**Technological affinity** *	56	72	65	50

*Note*.

* Measured with TA-EG (Karrer et al., 2009): score ranges from 19 (low affinity for technology) to 95

**Table 4:** Exemplary Tasks with Solution Paths for Different Devices

PWSLI (Tablet application)	PWSLI (Laptop web interface)	SLT (Tablet application)	SLT (Laptop web interface)
Task 1: Send a message to your Therapist XY. Ask when your next therapy session will take place.	Task 1: Send a message to your Therapist XY. Ask when your next therapy session will take place.	Task 1: Send a message to your patient XY. Announce when your next therapy session will take place.	Task 1: Send a message to your patient XY. Announce when your next therapy session will take place.
Solution paths: *Click “messages” in the top right -, select conversation with XY - click “your message” to open the text field - write message - click “send”*	Solution paths: *Home screen - click “my news” OR “Burger Button” - “my profile” - “own messages”*	Solution paths: *Click “messages” at the top right - select conversation with XY - click “your message” to open text field - write message - click “send”*	Solution paths: *Home screen - click “my news” OR “Burger Button” - “my profile” - “own messages”*

Task 2: You would like to practice before your next therapy session. Start the training and complete the exercises.	Task 2: You would like to practice before your next therapy session. Start the training and complete the exercises.	Task 2: Your video therapy session with your patient XY is about to start. Start the conference.	Task 2: Review the practice statistics of your patient XY. How often did they practice last week? When did they practice the most?
Solution paths: *Click “start training” - click “start tasks” - complete the exercises*	Solution paths: *Open HiSSS app - click “start task”*	Solution paths: *Press “start” next to the Name of patient XY*	Solution paths: *Click button - “my patients” - three dots in right column - “activities” - hover mouse - click “view HiSSS statistics”*

**Table 5:** Exemplary Checklist for Rating the Task Completion

Task	(1) Feasible without issues	(2) Feasible after some attempts /after short time	(3) Feasible with assistance /after long time	(4) Not feasible (assistance in every step)	Comments
**Send message**	X				
**Start training**	X				PWSLI only
**Open statistics**		X			
**Start video-call**			X		
**Write on whiteboard**			X		
**End video-call**	X				Not via ‘*end call*,’ but by exiting training mode
**Change settings**				X	Setting option only found in the main menu, not in training mode
**Change operator mode**		X			SLTs only

**Table 6:** Results of the Structured Observation after Consensus of the Two Raters

	P1	P2	P3	P4	P_Median_	T1	T2	T3	T4	P_Median_
**Send message**	3	3	3	2	3.0	1	1	1	1	1.0
**Start training**	2	2	2	1	2.0					
**Open statistics**	1	2	4	1	1.5	X	2	X	1	1.5
**Start video-call**	1	1	4	3	2.0	3	2	3	**2**	2.5
**Write on whiteboard**	**3**	3	4	4	3.5	1	1	3	1	1.0
**End video-call**	1	1	4	4	2.5	1	1	1	1	1.0
**Change settings**	1	1	4	3	2.0	1	X	4	X	2.5
**Operator handover**						3	2	2	2	2.0

**Median**	1.0	2.0	4.0	3.0		1.0	1.5	2.5	1.0	

*Note.* P: person with speech and language impairment, T: therapist; 1 = feasible without any issues; 2 = feasible after some attempts/ a short period of time; 3 = feasible with assistance; 4 = not feasible (assistance in every step), X = task not executed

**Table 7:** Key Results of the Focus Group

Main Categories	Key Findings
Facilitators	– Motivational character of the system – Variety of exercises – Customizability
Barriers	– Disorientation – Overlapping windows – Mistakes in automatic speech recognition
Suggestions for improvement	– More support options – Consistent use of terminology – More intuitive user flow – More precise descriptions of tasks
Future (Non-)Use	– Integration into telepractice context – Useful supplement for independent training
Training & Support	– Need for familiarization and good instructions – Combination of specific and general support
Participation (Only voiced by P)	– Positive feeling toward being involved in the development process – Option for acting as a multiplier in the community – Wish for reoccurring exchange

## Footnotes

1 HiSSS is the abbreviation of the project “Hybrid and interactive speech and language therapy after stroke” funded by the Federal Ministry of Sciences, Technology and Space. It does not exclude people with speech and/or language impairment associated with neurological disorders.
